# Highly Linear and Wide Non-Resonant Two-Degree-of-Freedom Piezoelectric Laser Scanner

**DOI:** 10.3390/s22114215

**Published:** 2022-06-01

**Authors:** Takashi Ozaki, Norikazu Ohta, Motohiro Fujiyoshi

**Affiliations:** Toyota Central R&D Labs. Inc., Nagakute 480-1192, Aichi, Japan; ohtan@mosk.tytlabs.co.jp (N.O.); fujiyoshi@mosk.tytlabs.co.jp (M.F.)

**Keywords:** piezoelectric actuator, scanning mirror, PIN-PMN-PT, compliant mechanism

## Abstract

Laser scanners with mechanically driven mirrors have exhibited increasing potential for various applications, such as displays and laser radar. Resonant scanners are the predominantly used scanners; however, non-resonant scanners are required for applications where point-to-point driving is desirable. Because a non-resonant drive cannot amplify the drive angle owing to the resonance phenomenon, high values are difficult to achieve for the main performance metrics of the scanners: mirror area, drive angle, and operating frequency. In this paper, we present a two-axis scanner with a piezoelectric actuator made of a piezoelectric single-crystal Pb(In_1/2_Nb_1/2_)O_3_-Pb(Mg_1/3_Nb_2/3_)O_3_-PbTiO_3_ as the actuation force source. The scanner contains a circular mirror with a diameter of 7 mm and achieves an average static mechanical deflection angle amplitude of 20.8° in two axes with a resonant frequency of 559 Hz. It is equipped with a transmission mechanism that can decouple each axis to achieve high linearity; in our study, the nonlinearity error was less than 1°.

## 1. Introduction

Movable mirrors with a wide scanning range are expected to have a wide range of applications, such as for use in laser scanners for projective displays [1,2,3,4], laser radar [5,6,7], optical communications [8,9], and laser machining [10,11]. Numerous scanners use a resonant drive as a simple method to increase the scanning angle amplitude, a fact which has been introduced in many papers. Conversely, a non-resonant drive allows point-to-point scanning, although it is difficult to achieve a large scanning angle amplitude. This point-to-point scanning capability is desirable for several applications, such as for laser radar measurement, free-space optical communication, and laser machining. Therefore, in this study, we focused on the non-resonantly driven scanners.

In general, the area of the mirror, search angle, and operating frequency are the main performance indicators of a laser scanner. For example, the measurement accuracy of time-of-flight (ToF) laser radar systems depends on the amount of light detected. Therefore, a large mirror area is useful for collecting more reflected light and increasing the signal-to-noise ratio. The scanning angle is directly related to the viewing angle. The operating frequency is the frame rate. To date, large, heavy, and costly electromagnetic motor-driven rotating polygon mirrors [12] have been commonly used. Thus, a smaller and cheaper actuator is desirable for the realization of this type of mirror, and a spring-supported scanning mirror has been considered as an alternative to the rotating polygon mirror. Electrostatic force [13,14,15,16], electrothermal force [17,18,19,20], electromagnetic force [21,22,23,24,25,26], and piezoelectric force [27,28,29,30,31,32] have been studied as the driving principles of the mirrors. Among these, electrostatic, electromagnetic, and piezoelectric drives are promising based on their response speed. For example, the electrostatically driven scanner of Mirrorcle Inc. contains a large mirror and a wide deflection angle (a mechanical deflection angle of 10.4° for a 5 mm circular mirror) [16]. In addition, an electromagnetic scanner developed by a research team at Sogang University contains an 8 mm square mirror and a deflection angle of 8.0° [25]. In the literature pertaining to the piezoelectric drive, scanners with stacked actuators have been reported as commercial products [30]. Generally, these scanners have small scanning angles and high resonance frequencies. To solve the drawback of the large power consumption caused by the large capacitance of the stacked actuators, scanners with bimorph/unimorph actuators have been researched. For example, the research group of Harbin Institute of Technology achieved a 0.09° scanning angle and a high resonance frequency of 356 Hz in a biaxial scanner with a bimorph actuator [31]. In our previous paper, we reported on a piezoelectric-driven scanner with unimorph actuators based on a piezoelectric single-crystal material Pb(In_1/2_Nb_1/2_)O_3_-Pb(Mg_1/3_Nb_2/3_)O_3_-PbTiO_3_ (PIN-PMN-PT), which has a large piezoelectric constant and can be easily fabricated [32]. Using a sandwich structure of thin titanium and polyimide, a lightweight and compliant transmission mechanism was created, and a mechanical scanning angle of approximately 20° was achieved with a 6 mm square mirror. However, the resonant frequency was low at approximately 100 Hz, and there was a strong nonlinearity between the driving voltage and the displacement angle.

Therefore, we designed a new transmission with four actuators and two decoupled axes. This new design can achieve a high frequency and large deflection angles (559 Hz and 20.8°, respectively), as well as high linearity. This performance is superior to that of conventional non-resonantly driven two-axis scanners and can increase the advantages of piezoelectric scanning mirrors.

## 2. Materials and Methods

### 2.1. Design of Developed Laser Scanner

**Figure 1:**
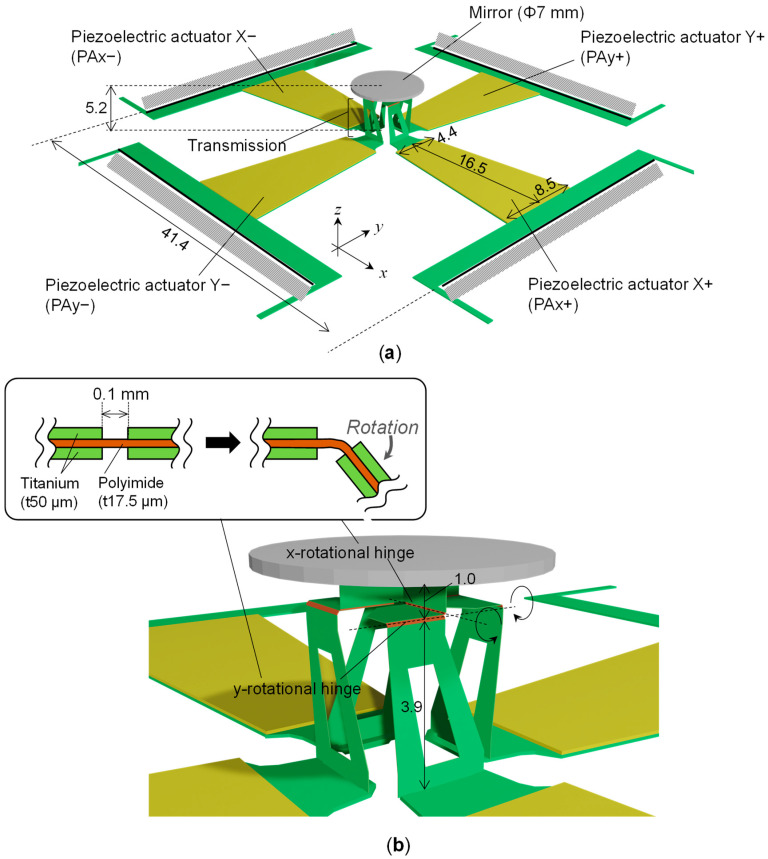
Structure of the developed scanner: (**a**) overall view and (**b**) close-up view of the transmission mechanism.

Figure 1a shows the overall view of the scanner, which consists of four actuators arranged in four directions, with a circular mirror (7 mm in diameter) in the center. The actuators on the positive and negative sides of the *x*-axis are referred to as piezoelectric actuator X+ (PAx+) and piezoelectric actuator X− (PAx−), respectively. Similarly, the actuators in the *y*-axis direction are referred to as piezoelectric actuator Y+ (PAy+) and piezoelectric actuator Y− (PAy−), respectively. The actuators are unimorphs, which consist of a piezoelectric layer attached to an elastic layer. In this study, the single-crystal piezoelectric material PIN-PMN-PT was used as the piezoelectric material, and titanium was used as the elastic layer. The thicknesses were 0.10 mm and 0.13 mm, respectively. The planar shape of the unimorph was trapezoidal with a height of 16.5 mm, a top edge of 4.4 mm, and a bottom edge of 8.5 mm. There was a transmission between the actuator and mirror. An enlarged view of the transmission is shown in Figure 1b. It was made of a layer of polyimide (17.5 μm thick) sandwiched between two layers of titanium (50 μm thick). As shown in the upper part of Figure 1b, there were areas where titanium had been removed and only polyimide was used as the compliant hinge. Each actuator and mirror were connected via a serial combination of the *x*- and *y*-axis rotational hinges. In a previous study, the resonant frequency was low because of the large distance between the heavy mirror and the center of rotation. In this study, the distance between the mirror and the center of rotation was reduced to 1 mm to increase the resonant frequency.

**Figure 2:**
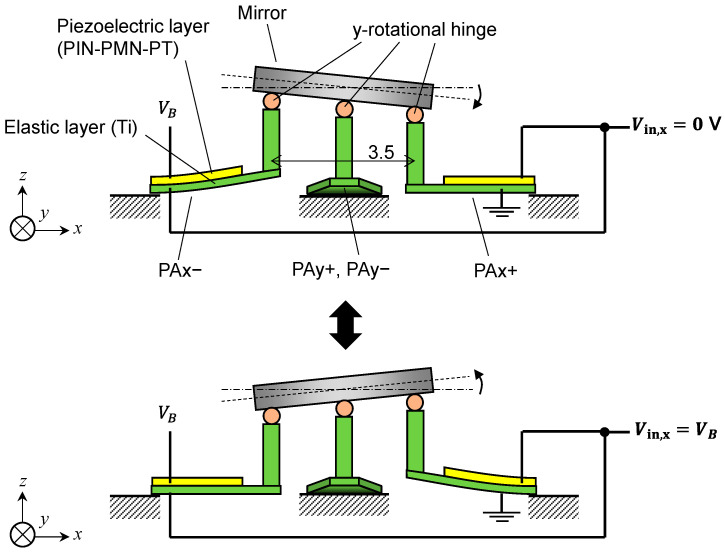
Principle of the scanning operation.

Figure 2 shows the actuation principle. Three voltage inputs are required to drive this scanner: there are variable voltage inputs $V_{\mathrm{in},x}$ and $V_{\mathrm{in},y}$ to drive the *x*- and *y*-axes, respectively, and a fixed bias voltage $V_{B}$. The lower electrode (elastic layer side) of PAx+ is grounded, and a constant bias voltage $V_{B}$ is applied to the upper electrode (piezoelectric layer side) of PAx−. When $V_{\mathrm{in},x}=0$ V, the potential difference applied to PAx+ is 0 V; therefore, no displacement occurs in PAx+. Conversely, because the potential difference at PAx− is $V_{B}$, the piezoelectric actuator PAx− warps upward. This lifts the edge of the mirror near PAx− and rotates it in a positive direction around the *y*-axis, while when $V_{\mathrm{in},x}=V_{B}$, the mirror rotates in a negative direction around the *y*-axis. By continuously changing $V_{\mathrm{in},x}$, the deflection angle of the mirror can be controlled. When $V_{\mathrm{in},x}=V_{B}/2$, the mirror is horizontal (the tilt angle is zero). In this case, only the y-rotational hinges act as hinges in the mechanism, not the x-rotational hinges. The motion by PAx+/− is not transmitted to the orthogonal actuators PAy+/−, owing to the y-rotational hinges. This allows us to decouple the drive of each axis. Because this mechanism is symmetrical in the *x*- and *y*-axes, the above explanation is the same when actuating PAy+/−.

**Figure 3:**
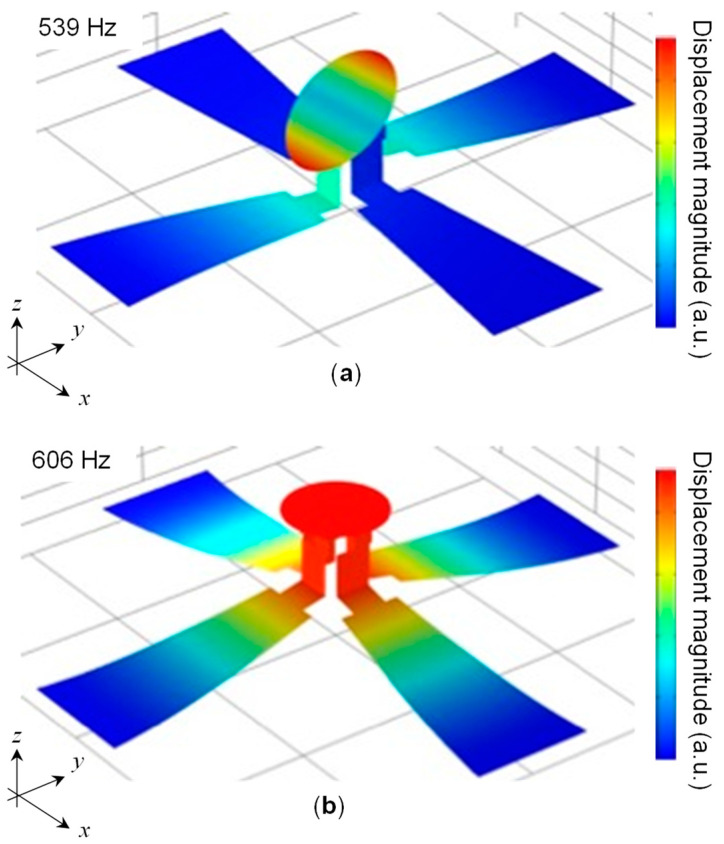
Result of the modal analysis: (**a**) First and (**b**) second resonant mode shapes.

**Figure 4:**
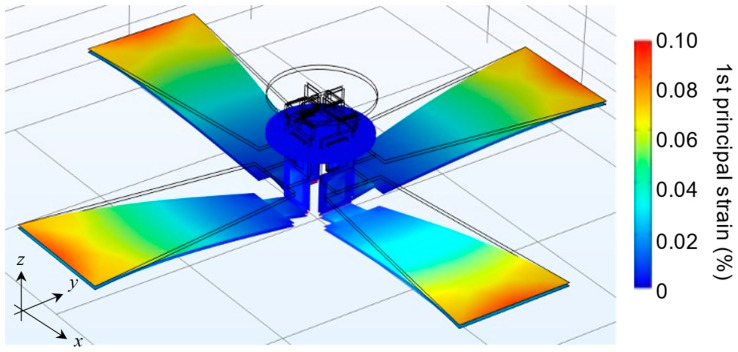
Result of the shock analysis: deformation shapes and strain contour plots when the maximum first principal strain was observed for an acceleration of 3000 G along the *z*-axis applied for 0.3 ms.

Finite element analysis (FEA) was used to analyze the characteristics of the design. Because the scanner is made entirely of thin plate structures, a shell model was used for the FEA modeling. Please refer to the Appendix A for the detailed material properties. The software used was COMSOL Multiphysics (COMSOL Inc., Burlington, MA, USA). Figure 3 shows the results of the resonance frequency analysis. The tilting motion of the mirror was the first mode with a frequency of 539 Hz. The second mode was an up-and-down vibration with a frequency of 606 Hz. The tilt angle of the mirror when voltage was applied was 0.136°/V, as determined by a linear static analysis. From this analysis, the maximum first principal strain in the piezoelectric material at a drive voltage of 140 V (the maximum voltage in the experiment) was calculated to be 0.16%. Since the fracture strain of a PIN-PMN-PT of similar dimensions is approximately 1.2% [33], this design was determined to have a sufficient margin. In addition, a shock load analysis was conducted in accordance with MIL-STD-883E [34], in which an acceleration of 3000 G was applied for 0.3 ms. Figure 4 shows the deformation shape and strain distribution at the moment of maximum strain. In this case, a maximum first principal strain of 0.11% occurred at the fixed end of the actuator; this value is sufficiently smaller than the fracture strain.

### 2.2. Fabrication

**Figure 5:**
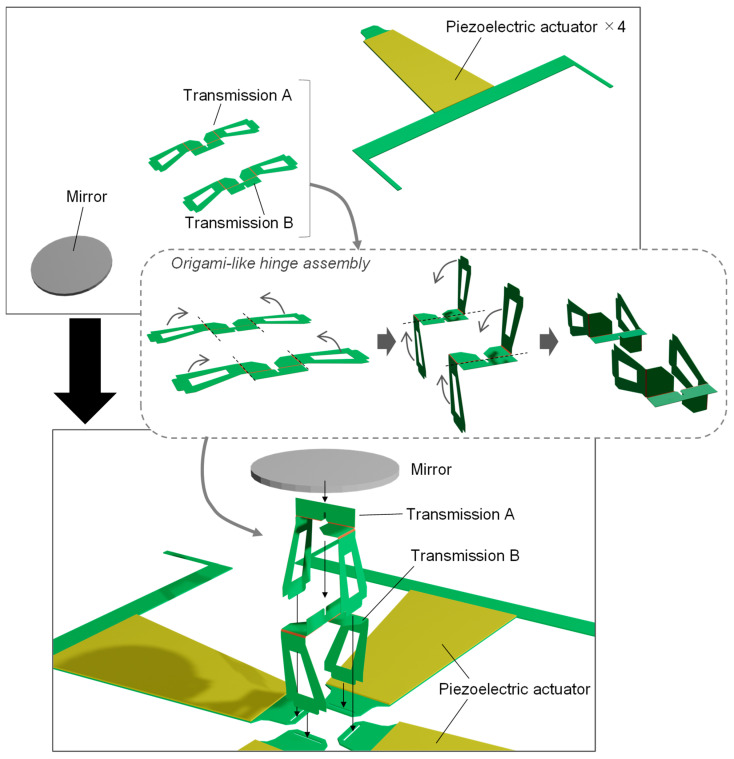
Assembly of the developed scanner.

The scanner consists of three main parts: a mirror, transmission, and piezoelectric actuator (upper part of Figure 5). The mirror was constructed by laser cutting a 300-μm-thick silicon wafer. The transmission is a laminated structure of titanium and polyimide, as described above. It was fabricated by bonding pre-patterned titanium and polyimide layers via thermocompression bonding. A detailed fabrication process is reported in [35]. The piezoelectric actuators were fabricated by bonding PIN-PMN-PT and the titanium layers with an epoxy resin (Quick 5; Konishi Co., Ltd., Osaka, Japan), and PIN-PMN-PT with an Au electrode was manufactured by TRS Technologies, Inc. (State College, PA, USA). Four piezoelectric actuators, two transmission parts, and a mirror were assembled, as shown in the lower part of Figure 5. Here, the hinge components, fabricated in a planar form, are assembled into a three-dimensional form, similar to origami, as shown in the middle portion of the figure. A UV-curable adhesive (Loctite 4305; Henkel AG & Company, Düsseldorf, Germany) was utilized to join the parts.

### 2.3. Evaluation

**Figure 6:**
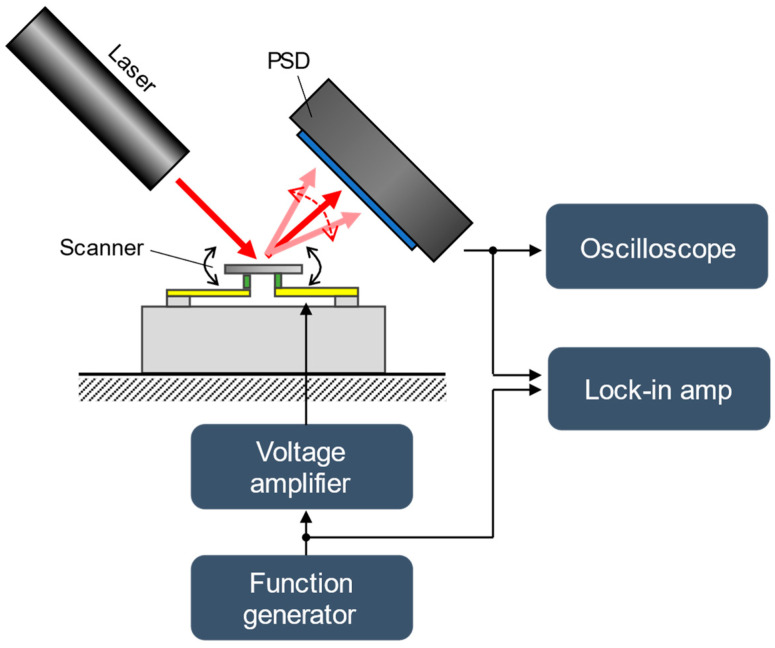
Schematics of the evaluation setup.

The evaluation system is shown in Figure 6. A red laser beam (37-029; Edmund Optics Corp., Barrington, NJ, USA) was utilized to irradiate the circular mirror surface from an angle. The position of the reflected laser was measured using a position sensitive detector (PSD) (PDP90A; Thorlabs, Newton, NJ, USA), and the tilt angle of the mirror was calculated from the position of the reflected light. A combination of a waveform generator (4054 B; BK Precision, Yorba Linda, CA, USA) and multichannel voltage amplifier (HJPZ-0.3P×3; Matsusada Precision Inc., Kusatsu, Japan) was used to drive the piezoelectric actuator. A lock-in amplifier (HF2LI, Zurich Instruments AG, Zurich, Switzerland) was used to measure the frequency response. An oscilloscope (4824A; Pico Technology, Cambridgeshire, UK) was used to record the mirror angle, such as when measuring the voltage-angle characteristics.

## 3. Results and Discussion

**Figure 7:**
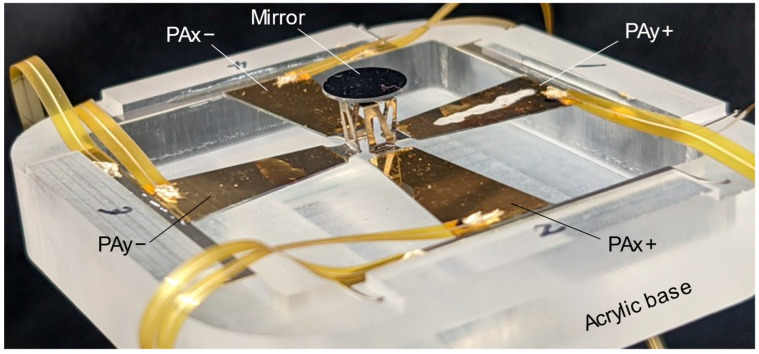
Photograph of the fabricated scanner.

Figure 7 shows an external view of the fabricated scanner. The root of the piezoelectric actuator was glued to an acrylic base. A flexible printed circuit cable was used as the wiring, and silver paste was used to connect the cable to the piezoelectric actuator.

**Figure 8:**
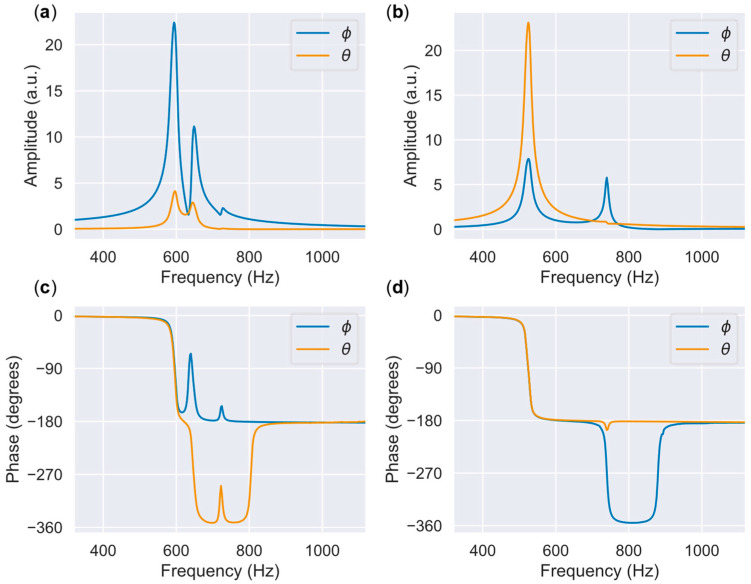
Frequency response: Frequency-amplitude characteristics under (**a**) *x*-axis actuation and (**b**) *y*-axis actuation; frequency-phase characteristics under (**c**) *x*-axis actuation (PAx+/−) and (**d**) *y*-axis actuation (PAy+/−).

**Figure 9:**
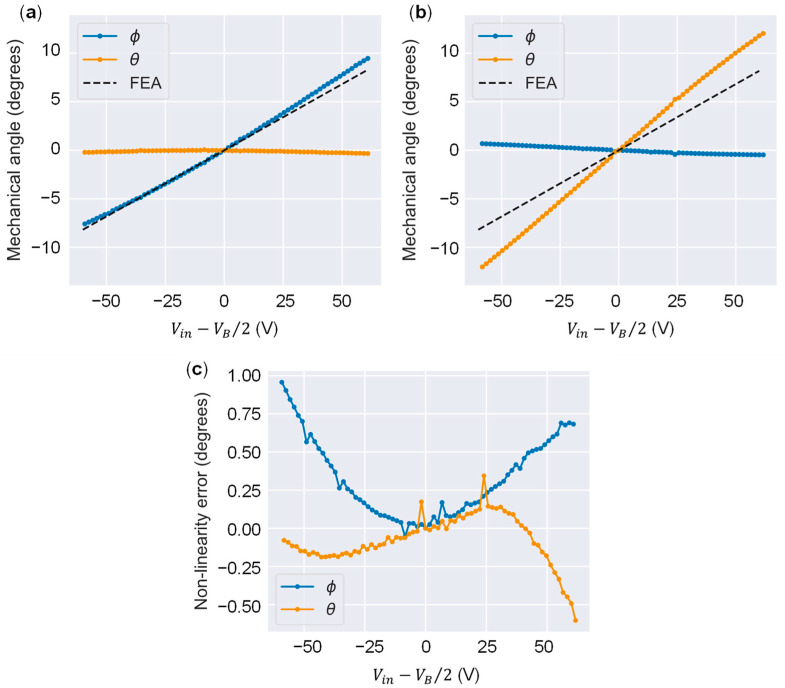
Voltage-angle characteristics: (**a**) *x*-axis and (**b**) *y*-axis actuations and (**c**) nonlinearity error.

**Figure 10:**
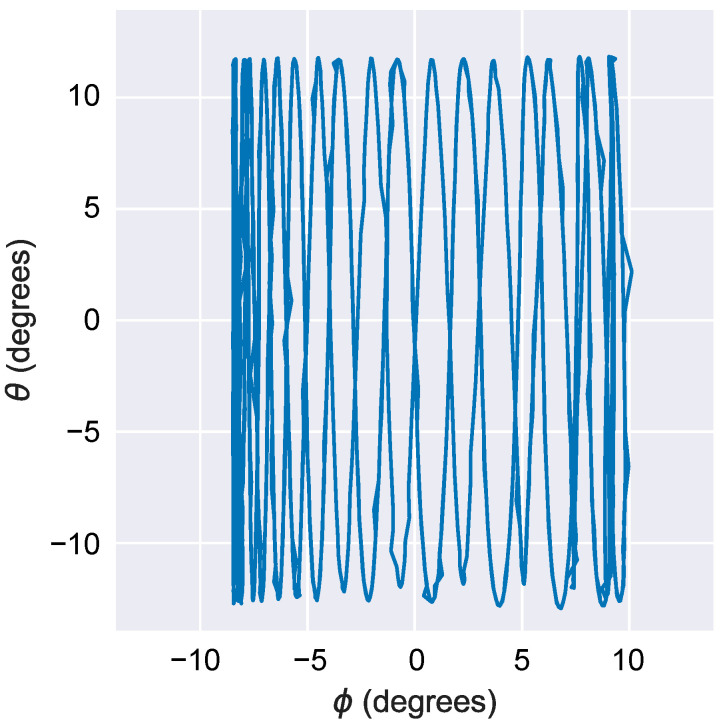
Two-axis actuation demonstration.

Figure 8 shows the frequency response characteristics. In this experiment, a frequency sweep over a certain range of a sine wave of an amplitude of 1 V was conducted, and the amplitude and phase of the mirror angle were measured. The mirror rotation angle around the *x*-axis is defined as Φ, and the mirror rotation angle around the *y*-axis is defined as θ. Figure 8a shows the amplitudes of Φ and θ when a sinusoidal drive voltage is applied to PAx+ and PAx−. Figure 8b shows the amplitudes of Φ and θ when PAy+ and PAy− are also driven. Figure 8c,d show the phase graphs when PAx+/− and PAy+/− are driven, respectively. The resonant frequency is the frequency at which the maximum amplitude is reached, and the phase is −90°. The resonant frequencies around the *x*-axis (Φ) and *y*-axis (θ) are 594 Hz and 524 Hz, respectively. Considering an amplification ratio of +1% or less as the quasi-static drive range, the operating frequency range is 1/10 of the resonance, 52–59 Hz, with the quality factor of approximately 20. This is similar to the practical value for the display and radar refresh rates. Figure 9a,b shows the mirror angle versus the applied voltage. The FEA results are represented by dotted lines. The bias voltage $V_{B}$ was set to 140 V, and the drive voltage $V_{in}$ was varied between 0 and 140 V. The mirror becomes horizontal when $V_{in}=V_{B}/2$. When PAx+/− is driven, the range of change in Φ is 17.2°, and when PAy+/− is driven, the range of change in θ is 24.4°. The average angle range was 20.8°; therefore, a large amplitude motion was achieved. The tilt angle per unit voltage was 0.149°/V, which is in good agreement with the FEA result of 0.136°/V. The resonant frequency also matched the experimental result and the FEA (559 Hz on average for the two axes and 539 Hz, respectively), indicating that the structure was fabricated as designed. In this experiment, the unimorphs were assembled by hand, and variations in the amount of adhesive applied during assembly possibly caused this error. The difference in resonant frequency is estimated to be due to the difference in stiffness caused by the assembly error of the unimorphs. There is a correlation between the resonance frequency $f_{n}$, the spring constant $K_{u}$ of the unimorph, its thickness $t_{u}$, and its length $L_{u}$ given by the expression $f_{n}\propto K_{u}^{1/2}\propto t_{u}^{3/2}L_{u}^{-3/2}$. Thus, the 13% difference in $f_{n}$ corresponds to an error of 8.5% in terms of $t_{u}$ and $L_{u}$. The difference in the tilt angle per unit voltage is smaller on the *x*-axis where $f_{n}$ is higher, i.e., in the direction where $K_{u}$ is estimated to be higher. Therefore, a primary cause seems to be that a large $K_{u}$ inhibits the actuation. Furthermore, the variation in the piezoelectric constants would combine to produce asymmetry in the tilt angle characteristics. To improve the asymmetry of the characteristics, the manufacturing and assembly process of unimorphs should be made uniform. For the ranges of crosstalk, the change in θ when driving PAx+/− was 0.59°, and the change in Φ when driving PAy+/− was 1.47° in the drive voltage range of −70 to +70 V. The change in crosstalk when driving PAy+/− was larger; however, it was less than 6% of the main drive angle range. The decoupling mechanism realized minimal crosstalk. If the geometry is ideally symmetrical, coupling should not occur. The main cause of coupling is assumed to be symmetry breaking during the transmission assembly; because the transmission is assembled manually, the process is prone to errors. The insertion part for attaching the transmission to the actuator tip has a margin of 0.4 mm of width to facilitate fitting. Therefore, a maximum error of ±0.2 mm can occur in the assembly position. Because the distance from the hinge to the center of mirror rotation is 1.75 mm, an orthogonality error could produce a coupling of 0.2 mm/1.75 mm = 11.4% at most. Figure 8c shows the nonlinearity (deviation from a linearly fitted line) of the voltage-mirror angle. The nonlinear error is small, less than 1°, indicating that high linearity has been achieved by this design. A 2D scanning demonstration result using this scanner is shown in Figure 10. Here, PAx+/− was driven by a 1 Hz sine wave and PAy+/− was driven by a 20 Hz sine wave. We attempted to draw a 1:20 Lissajous figure. The voltage amplitude was set to 140 V for both axes. As shown in the figure, the Lissajous figure was drawn with little distortion, and it was confirmed that each axis maintained a high independence, even when the two axes were driven simultaneously.

**Figure 11:**
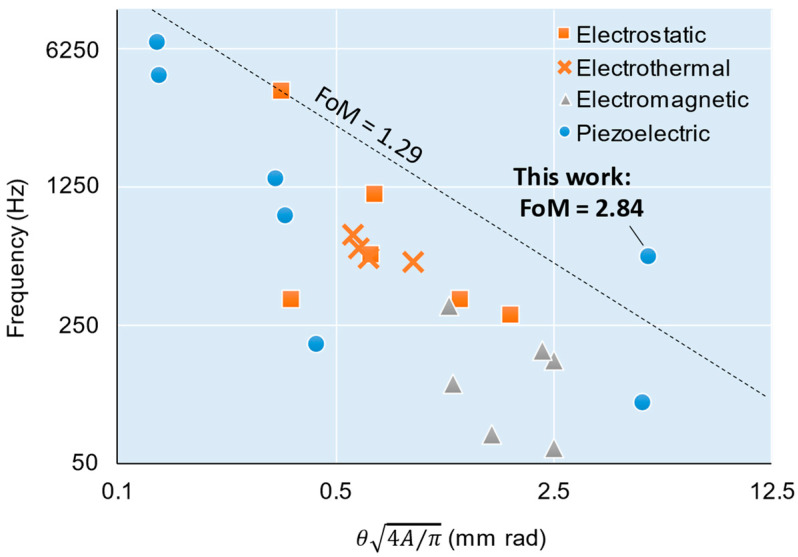
Comparison of 2-DoF quasi-static scanners.

Next, the level of achievement for this scanner was compared to those of previous scanners. Here, we adopted the figure-of-merit (FoM) proposed in a review report [36] as the measure of scanner performance. The FoM is calculated as $\theta \sqrt{4A/\pi }f$, where θ, A, and f are the optical scanning angle amplitude, area of the mirror plate, and resonant frequency, respectively. Table 1 lists the performances and FoMs of the previously reported two-axis non-resonant scanners. Figure 11 shows a graph of $\theta \sqrt{4A/\pi }$ on the horizontal axis and f on the vertical axis. As listed in the table, the new scanner achieved a higher FoM than that of conventional scanners. In particular, a large deflection angle and a high resonant frequency are achieved in a large-area mirror, which are outstanding features. These features are superior to commercial piezoelectric scanners in some respects because commercial scanners with commonly used stacked piezoelectric elements currently have scan angles of only a few degrees at most. For example, S-335 (Physik Instrumente GmbH & Co. KG, Karlsruhe, Germany) has a scan angle of 2°, a resonant frequency of 1.6 kHz, and a FoM of 0.70. PSH25 (Piezosystem Jena, Jena, Germany) has a scan angle of 1.1°, a resonant frequency of 1.4 kHz, and a FoM of 0.35. In addition, for piezoelectric actuators that are capacitively loaded, large capacitance leads to the drawback of requiring large power. Stacked piezoelectric actuators have capacitance as large as several μF (e.g., 6.2 μF and 1.64 μF for the two products mentioned above, respectively). In contrast, the capacitance of our scanner is two orders of magnitude smaller at 25 nF, owing to its single layer actuator. In other words, this scanner exhibits the advantage of low power consumption.

## 4. Conclusions

In this study, we developed a piezoelectric-driven laser scanner with a size of 41.4 mm × 41.4 mm × 5.2 mm and a mirror diameter of 7 mm, which achieves a large amplitude of 20.8° on average and a high resonance frequency of 559 Hz. A major feature of this scanner is its large scan angle, which makes it particularly suitable for applications where a wide field-of-view is important, such as point-to-point ToF radars and displays. Further, our scanner can potentially be applied as a compact laser processing machine for medical use in clinics. Future tasks include the addition of an angle-detection function and durability verification. The main limitation of this scanner is the cost of the piezoelectric material used (PIN-PMN-PT). The potential to replace the material with Pb(Zr_1−x_Ti_x_)O_3_ (PZT) ceramics, which is cheaper, should be considered. In addition, the possibility of miniaturization could be evaluated. For example, if the unimorphs that are currently arranged in a crisscross pattern were arranged in a tetraskelion pattern, the layout would be denser, and the size would become smaller.

## Figures and Tables

**Table 1:** Comparison of 2-DoF mechanical quasi-static scanners.

Ref.	Actuation Principle	Mirror Area (mm^2^)	Deflection Angles (Degrees)	Resonant Frequencies (Hz)	FoM (mm·rad·kHz)
[13]	Electrostatic	0.50	12.0	3800, 3900	1.29
[13]		2.0	12.0	670, 1600	0.76
[14]		0.79	12.4, 8.2	350, 320	0.12
[16]		19.6	10.4	278	0.50
[16]		32.2	5.56, 5.64	339, 337	0.42
[16]		44.2	2.49, 2.49	559, 557	0.37
[17]	Electrothermal	0.52	20.0	690, 740	0.41
[18]		0.20	2.0	12,800	0.45
[19]		0.81	18.0	550	0.35
[20]		5.0	7.5, 6.0	615	0.37
[17]		0.25	51.0, 39.5	170, 870	0.46
[21]	Electromagnetic	4.9	30.0, 23.0	160, 210	0.43
[22]		9.0	10.0	130, 120	0.15
[23]		16.0	16.0	160, 170	0.42
[24]		13.4	8.0	240, 390	0.36
[25]		64.0	7.85, 8.10	60	0.15
[26]		0.11	120	70	0.11
[27]	Piezoelectric	1.2	2.1, 1.8	195	0.0082
[28]		0.79	9.3	1370	0.44
[29]		0.50	12.5	900	0.087
[30]		707	0.13	4578	0.63
[31]		314	0.09	356	0.023
[32]		36.0	21.6, 19.4	89, 111	0.48
This work		38.5	20.8 (in average)	559 (in average)	2.84

## Data Availability

The data presented in this study are available upon request from the corresponding author.

## Appendix A. FEA Model Parameters

Because piezoelectric actuators and transmission mechanisms are composed of laminated materials, the physical properties input to the shell model must consider the layered structure. The material property values for the materials used in the FEA are listed in Table A1. The full parameter matrix of PIN-PMN-PT is reported in [37]. For example, the piezoelectric actuator consists of three layers: titanium, PIN-PMN-PT, and an epoxy resin that bonds these layers. The bending stiffness of the laminates can be calculated as follows:

**Table A1:** Material properties for FEA.

Material	Property	Value
PIN-PMN-PT	Young’s modulus	14.3 GPa ^1^
	Density	8000 kg/m^3^
	Piezoelectric coefficient	−1156 pm/V
Titanium	Young’s modulus	116 GPa
	Density	4500 kg/m^3^
Polyimide	Young’s modulus	9.0 GPa
	Density	1500 kg/m^3^
Epoxy	Young’s modulus	5.0 GPa
	Density	1500 kg/m^3^
Silicon	Young’s modulus	160 GPa
	Density	2330 kg/m^3^

^1^ Given by the reciprocal of $s_{22}^{E}$ value of PIN-PMN-PT [37].

The position of the neutral axis, $z_{c}$, can be calculated as follows:

(A1) $$z_{c}=\frac{\sum_{i}E_{i}t_{i}z_{i}}{\sum_{i}E_{i}t_{i}},$$

where, $E_{i}$ is the Young’s modulus of the *i*-th layer. The unimorph has a three-layer structure, where *i* = 1, 2, and 3; these are titanium, epoxy resin, and piezoelectric PIN-PMN-PT, respectively. Similarly, $t_{i}$ and $z_{i}$ are the thickness and position in the thickness direction, respectively. Based on this, the bending stiffness *s* per unit width can be obtained using the following equation:

(A2) $$s=\underset{i}{\sum }E_{i}\left(\frac{t_{i}^{3}}{12}+t_{i}{\left(z_{i}-z_{c}\right)}^{2}\right).$$

From the following equation, the bending moment per unit width, m, due to the piezoelectricity per unit voltage V can be expressed [38]

(A3) $$m=E_{3}d_{pz}\left(z_{3}-z_{c}\right)V$$

where $d_{pz}$ is the piezoelectric coefficient of PIN-PMN-PT.
